# Supplementary material for: Resolving the full spectrum of human genome variation using Linked-Reads
Source: Genome Res. 2019 Apr;29(4):635–45. doi: 10.1101/gr.234443.118 (PMC6442396; doi:10.1101/gr.234443.118)
Supplement: Supplemental Material [file supp_gr.234443.118_Supplemental_longranger-2.2.zip › longranger-2.2.tar.bz2!/lr2.2-public/lib/stan/cmdstan/stan/doc/api/html/resize_8hpp_source.html]

Stan: src/stan/math/matrix/resize.hpp Source File


|  |  |
| --- | --- |
|  | Stan  2.5.0  probability, sampling & optimization |


- Main Page
- Related Pages
- Namespaces
- Classes
- Files

- File List
- File Members

All Classes Namespaces Files Functions Variables Typedefs Enumerations Enumerator Friends Macros Pages

- src
- stan
- math
- matrix

resize.hpp

Go to the documentation of this file.

1 #ifndef STAN\_\_MATH\_\_MATRIX\_\_RESIZE\_HPP

2 #define STAN\_\_MATH\_\_MATRIX\_\_RESIZE\_HPP

3

4 #include <vector>

5 #include <stan/math/matrix/Eigen.hpp>

6

7 namespace stan {

8  namespace math {

9

10  namespace {

11

12  template <typename T>

13  void resize(Eigen::Matrix<T,Eigen::Dynamic,Eigen::Dynamic>& x,

14  const std::vector<size\_t>& dims,

15  size\_t pos) {

16  x.resize(dims[pos],dims[pos+1]);

17  }

18

19  template <typename T>

20  void resize(Eigen::Matrix<T,Eigen::Dynamic,1>& x,

21  const std::vector<size\_t>& dims,

22  size\_t pos) {

23  x.resize(dims[pos]);

24  }

25

26  template <typename T>

27  void resize(Eigen::Matrix<T,1,Eigen::Dynamic>& x,

28  const std::vector<size\_t>& dims,

29  size\_t pos) {

30  x.resize(dims[pos]);

31  }

32

33  template <typename T>

34  void resize(T /\*x\*/,

35  const std::vector<size\_t>& /\*dims\*/,

36  size\_t /\*pos\*/) {

37  // no-op

38  }

39

40  template <typename T>

41  void resize(std::vector<T>& x,

42  const std::vector<size\_t>& dims,

43  size\_t pos) {

44  x.resize(dims[pos]);

45  ++pos;

46  if (pos >= dims.size()) return; // skips lowest loop to scalar

47  for (size\_t i = 0; i < x.size(); ++i)

48  resize(x[i],dims,pos);

49  }

50

51  }

52

62  template <typename T>

63  inline void resize(T& x, std::vector<size\_t> dims) {

64  resize(x,dims,0U);

65  }

66

67  }

68 }

69 #endif

stan::math::resize

void resize(T &x, std::vector< size\_t > dims)

Recursively resize the specified vector of vectors, which must bottom out at scalar values...

**Definition:** resize.hpp:63

stan::math::dims

void dims(const T &x, std::vector< int > &result)

**Definition:** dims.hpp:13

Eigen.hpp


---

[ Stan Home Page ]

*© 2011–2014,
Stan Development Team.*
